# Antibiotic Usage in the COVID-19 Intensive Care Unit of an Infectious Diseases Hospital from Nord-Eastern Romania

**DOI:** 10.3390/medicina59040645

**Published:** 2023-03-24

**Authors:** Andrei Vâţă, Florin Manuel Roşu, Olivia Simona Dorneanu, Alina Elisabeta Lehaci, Ştefana Luca, Isabela Ioana Loghin, Ioana Diandra Miftode, Cătălina Mihaela Luca, Egidia Gabriela Miftode

**Affiliations:** 1 Department of Infectious Diseases, “Grigore T. Popa” University of Medicine and Pharmacy, 700115 Iasi, Romania; andrei.vata@umfiasi.ro (A.V.); isabelabegezsan@yahoo.com (I.I.L.); catalina_luca2006@yahoo.com (C.M.L.); emiftode@yahoo.co.uk (E.G.M.); 2 Department of Dento-Alveolar Surgery, Anesthesia, Sedation and Medical-Surgical Emergencies, “Grigore T. Popa” University of Medicine and Pharmacy, 700115 Iasi, Romania; 3 Microbiology Department, “Grigore T. Popa” University of Medicine and Pharmacy, 16 Universității Street, 700115 Iași, Romania; odorneanu@yahoo.com; 4 “Sfânta Parascheva” Infectious Disease Hospital of Iaşi, 700116 Iaşi, Romania; alinalehaci03@gmail.com; 5 Department of Plastic Surgery and Reconstructive Microsurgery, “St Spiridon” County Emergency Hospital, 700111 Iasi, Romania; stepyluca@yahoo.com; 6 Department of Radiology, “St. Spiridon” County Emergency Clinical Hospital, 700111 Iasi, Romania; diandramiftode98@yahoo.com

**Keywords:** COVID-19, antibiotic, resistance, inflammation

## Abstract

*Background and Objectives.* The intensive care unit (ICU), especially in an infectious disease hospital, is both an area with a high consumption of antibiotics (atb) and a “reservoir” of multidrug-resistant bacteria. We proposed the analysis of antibiotic therapy practices in such a department that treated, in conditions of a pandemic wave, patients with COVID-19 and its complications. *Materials and Methods.* This was a retrospective transversal study of 184 COVID-19 patients treated in the ICU of a regional infectious disease hospital of Iaşi, Romania, in a 3-month interval of 2020 and 2021. *Results.* All the included patients (Caucasians, 53% males, with a median age of 68 years, and a Charlton comorbidity index of 3) received at least one antibiotic during their stay in the ICU (43% also had antibiotics prior to hospital admission and 68% in the Infectious Diseases ward). Only 22.3% of the ICU patients had only one antibiotic. A total of 77.7% of them started with an association of two antibiotics, and 19.6% of them received more than three antibiotics. The most-used ones were linezolid (77.2%), imipenem (75.5%), and ceftriaxone (33.7%). The median atb duration was 9 days. No change in the number or type of atb prescription was seen in 2021 (compared to 2020). Only 9.8% of the patients had a microbiological confirmation of bacterial infection. A total of 38.3% of the tested patients had elevated procalcitonin levels at ICU admission. The overall fatality rate was 68.5%, with no significant differences between the two analyzed periods or the number of administered antibiotics. More than half (51.1%) of the patients developed oral candidiasis during their stay in the ICU, but only 5.4% had *C. difficile* colitis. *Conclusion.* Antibiotics were widely used in our ICU patients in the presence of a reduced microbiological confirmation of a bacterial co-infection, and were justified by other clinical or biological criteria.

## 1. Introduction

Bacteria have been living around and inside humans from the beginning of their evolution [1]. Some of them have pathogenic proprieties and are linked to several diseases. The discovery of antibiotic substances, about 100 years ago revolutionized modern medicine, saving countless lives. Nevertheless, bacteria have been fighting back, adapting, and developing resistance mechanisms to most known antibiotics (atb), threatening to drag medicinal progress back at least a century.

The World Health Organization recently recognized antibiotic resistance as one of the biggest threats to global health, food security, and development and also that the misuse and overuse of antimicrobials are the main drivers of the occurrence of drug-resistant bacteria [2]. Pathogens disproportionately infect critically ill patients, and the intensive care unit (ICU) often harbors multidrug-resistant bacteria [3]. Thus, the use of antibiotics in the ICU is widespread and the employment of broad-spectrum, newer substances is common.

The COVID-19 pandemic hit most healthcare systems around the world hard and fast. Despite being a viral infection, most patients with severe forms of the disease received antibiotics [4,5], especially at the beginning of the pandemic in general wards and ICUs. Recent data [6,7] show that about two-thirds of COVID-19 patients (hospitalized or community treated, of all ages) receive antibiotic prescriptions. In some low- and middle-income countries, self-administration of antibiotics for treating or preventing the SARS-CoV-2 infection was a common practice, and the number of excess doses of antibiotics sold (especially macrolides) was significant [6,8,9]. The effects of this widespread and likely unnecessary use of antibiotics in COVID-19 patients raised concerns regarding the negative effects on the bacterial antimicrobial resistance (AMR) rates, even from the beginning of the pandemic [10,11]. Disruption of antibiotic stewardship programs in some facilities due to overcrowding, a lack of resources, excessive prudence, or fear of the unknown were seen during this pandemic and could worsen the AMR [12] rates.

Despite this atb overuse, a higher rate of healthcare-associated infections have been reported during the COVID-19 pandemic [13,14] with a decreasing trend in co-infections due to community bacteria and an increase in MDRs. This could have been a consequence of hospital overcrowding, relaxation of atb stewardship protocols, the use of mechanical protection barriers (facial masks), or a combination of the above.

Although there are numerous studies regarding atb use in COVID-19 patients from the community or hospitalized non-ICU wards, data on what has happened in the dedicated intensive-care wards during the heights of the pandemic waves are less abundant. Our goal was to assess the antibiotic use in severe and critical COVID-19 patients hospitalized in the ICU ward of our infectious diseases hospital in Romania during a 3-month period in 2020 and 2021.

## 2. Materials and Methods

This was a retrospective transversal study of COVID-19 patients treated in the ICU of an infectious diseases hospital of Iasi, Romania. We included 184 patients: 63 consecutive adult (>18 years old) patients who were admitted between 01 October and 31 December of 2020 and 121 from the same period of 2021. In the first time interval, the regional peak of the alpha pandemic wave was reached (9799 new confirmed cases/day or 521.46 cases/million inhabitants) and in the second, the peak of the regional delta pandemic wave (18,863 new cases/day or 959.5 cases/million inhabitants) [15].

COVID-19 was confirmed by SARS-CoV-2 detection in nasopharyngeal swabs using RT-PCR (viral RNA extraction was executed with the Exiprep™ 96 Viral DNA/RNA Kit on the automatic Exiprep 48 BIONEER extractor (BIONEER Corporation, Daejeon, Republic of Korea) and the amplification and detection of specific genes (RdRp, N and E genes) was executed with the GeneFinder™ COVID-19 kit Plus RealAmp Kit on a Quantstudio5 APPLIED BIOSYSTEM analyzer (Thermo Fisher Scientific Inc., Waltham MA, USA). Demographic data, including age, sex, and clinical, microbiological, and therapeutical data were collected from the patients’ charts and using electronic databases. All patients included in the study were also examined using an imaging system (computerized tomography scans or chest radiography) and evaluated for blood inflammatory markers (RX Imola analyzer—Randox Laboratories Ltd., Crumlin, UK) and hematological parameters (leukocyte count, percentage of neutrophils, mean neutrophil count and platelet count, C reactive protein, serum IL6 levels and fibrinogen) using a Sysmex xn550 (XN-550; Sysmex Corporation, Kobe, Japan) fully automated bidirectional analyzer–fluorescence and flow cytometry. Bacterial antibiotic susceptibility was assessed by the disk diffusion method using EUCAST standards [16]. Bacteria were classified as multidrug-resistant (MDR), extensively drug-resistant (XDR), and pandrug-resistant bacteria (PDR) according to Magiorakos et al.’s international expert proposal [17]. We used the 2021 AWaRe classification [18] proposed by the World Health Organization (WHO) to stratify atb types and the Charlton score to assess comorbidities [19].

The study was conducted with full adherence to the international norms of medical ethics, as set out in the Declaration of Helsinki. The patients gave their informed written consent for the use of their medical data for research purposes. The study was approved by the “Sfanta Parascheva” Infectious Diseases Hospital of Iași Ethics Committee.

Statistical analysis was performed using the Analyze-it add-on for Microsoft Excel (Analyze-it Software, Ltd. v 1.73, Leeds, UK). Descriptive data are presented as absolute values, percentages, and means. Differences between groups were tested for statistical significance using unpaired T-Student and χ^2^ square tests. *p* < 0.05 was considered to indicate a statistically significant difference.

## 3. Results

The demographics and co-morbidities are summarized in Table 1. Most patients were over 65 years old (56%) with multiple associated diseases (median Charlton index of 3). A total of 33.7% of them have more than three co-morbidities.

Most of the patients (76.1%) were first admitted to the ID ward of our hospital and later transferred to the ICU, when their condition worsened. Forty patients were already intubated and mechanically ventilated at ICU admission. A total of 33 patients received only continuous positive airway pressure (CPAP) ventilation. Overall, 82.1% of patients were intubated and mechanically ventilated during their hospitalization; 84% of these patients died.

The patients were admitted to the ID ward between 1 and 10 days (median 5 days) after disease onset and to the ICU between 3 and 20 days (with a median 9 days) after the first COVID-19 symptoms appeared.

All 184 patients received antibiotics while in the ICU.

A total of 79 (43%) received antibiotics prior to hospitalization (39 with prescription from the family physician) and 126 (68.5%) received antibiotics while in the infectious diseases ward, before the admission in the ICU.

**Figure 1:**
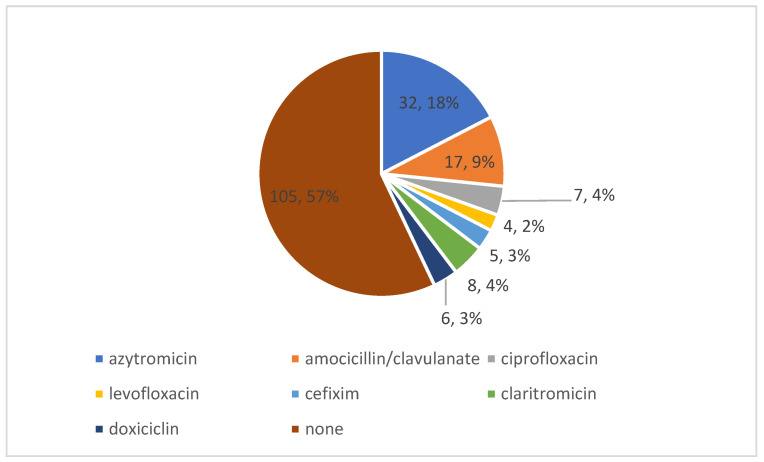
Antibiotics used before hospitalization.

The most commonly used antibiotic in ambulatory settings in our patients was Azithromycin, administered in 18% of cases (Figure 1).

**Figure 2:**
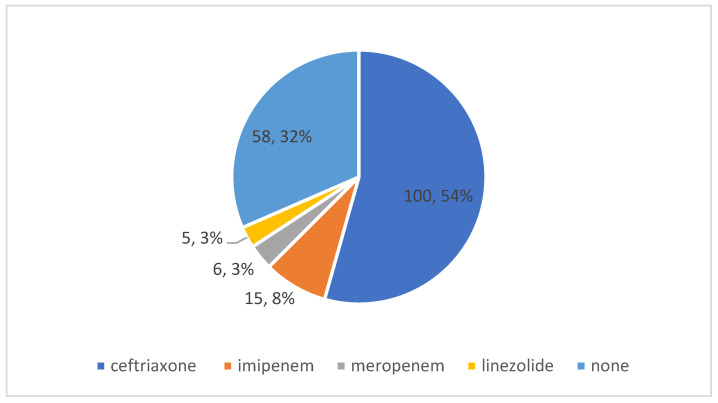
Antibiotics used in the ID ward.

During hospitalization in the ID ward, Ceftriaxone was the most-used antibiotic, administered in 54% of cases (Figure 2).

In the ICU, only 41 patients (22.3%) received antibiotics in monotherapy: 25 with imipenem-cilastatin, 13 with ceftriaxone, 1 with meropenem, 1 with piperacillin-tazobactam, and 1 with linezolid.

A total of 143 patients (77.7%) started with an association of 2 antibiotics, the most common being imipenem + linezolid (94 patients) targeting, in general, a respiratory co-infection. Other combinations used were: meropenem + linezolid (18 patients), meropenem + amikacin (12 patients), imipenem + vancomycin (9 patients), ampicillin/sulbactam + amikacin (6 patients), and ciprofloxacin + linezolid (4 patients).

No significant changes in the proportion of patients that received at ICU admission the association imipenem + linezolid was found between 2020 and 2021 patients: 35.7 vs. 38.5%, *p* = 0.63.

The duration of antibiotic treatment in the ICU varied between 1 and 31 days with a median of 9 days.

Changes in the antibiotic treatment of the ICU COVID-19 patients were found in 92 cases (50%). The causes of antibiotic changes are summarized in Table 2.

**Figure 3:**
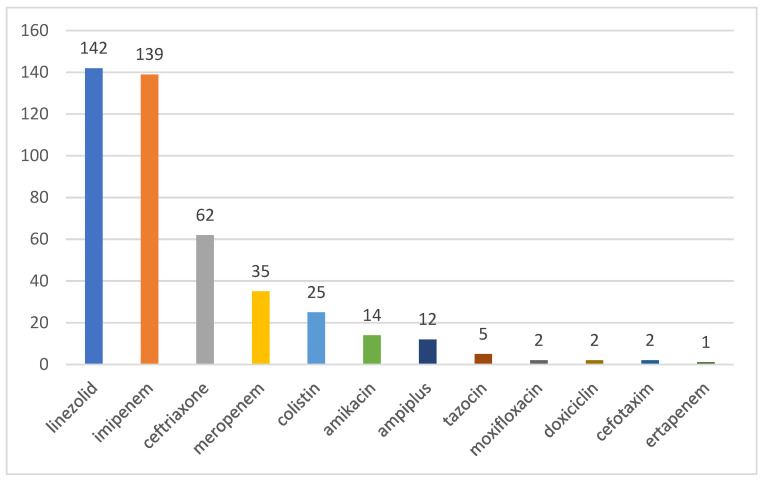
Antibiotics used in ICU COVID-19 patients.

Overall, the frequency of antibiotic use in our ICU patients is shown in Figure 3. Of them, three are included in the Access group of the AWaRe Classification (amikacin, ampicillin-sulbactam, and doxycycline), seven in the Watch group (Imipenem, Meropenem, ertapenem, Ceftriaxone, Cefotaxime, Moxifloxacin and piperacillin/tazobactam), and two in the Reserve group (Linezolid and Colistin).

During the whole duration of their COVID-19 infection, our patients received a median of two antibiotics (an average of 2.6); 19.6% of them received more than three (Table 3).

The average number of antibiotics used decreased only slightly in 2021 compared to 2020 patients (2.6 vs. 2.7, *p* = 0.56).

The overall fatality rate was 68.5%, with no significant differences between the two analyzed periods: 62.9% in 2020 and 71.3% in 2021 (*p* = 0.24). The rate of mortality in our study was higher in patients who received three or more antibiotics during their illness—74.2% vs. 63.2%—but the difference was not statistically significant (*p* = 0.1).

The number of prescribed antibiotics could have been influenced by the patients’ comorbidities, but our analysis showed no difference (a median Charlton score of 3, regardless of the number of antibiotics received).

A total of 97.8% of patients received treatment with dexamethasone (105 patients at a standard dose of 6 mg/day and the rest of the 79 patients at higher, immunosuppressive doses). A total of 63 patients also received treatment with Tocilizumab and 97 Anakinra. All had anti-coagulant medication.

The peripheral leukocyte count at ICU admission was higher than the upper normal limit in 88 patients (47.8%), with an average of 10,891/mm^3^; the neutrophils proportion was higher than normal in 75% of patients (average of 83.9%). A total of 25 patients associated thrombocytopenia at that moment. The average CRP was 118.2 mg/L and the fibrinogen level was 4.85 g/L. The ferritin level was measured in only 61.2% of patients, with an average of 1597 micrograms per liter. The IL-6 concentrations were determined in only 33 patients, with an average of 405.5 pg/mL. The procalcitonin level was assessed at ICU admission in 32.6% of patients; levels above normal were obtained in 38.3% of these cases.

None of the inflammatory markers from the moment of ICU admission were useful in predicting the outcome of the SARS-CoV-2 infection (Table 4).

A bacterial co-infection was clinically suspected in all patients admitted to the ICU. A total of 20.1% of patients developed a fever in the ICU. The localization of the bacterial co-infection was pulmonary (lobar pneumonia)—60 patients, urinary—15 patients, and involving skin and soft tissues—2 cases. Positive hemocultures were found in five patients.

Only 18 patients (9.8%) had a microbiological confirmation of bacterial infection: 5 patients with *Pseudomonas aeruginosa*, 3 patients with *A. baumanii*, 4 with *K. pneumoniae*, 2 with *S. aureus*, *E coli*, *Proteus* spp. Four of the five *P. aeruginosa* and two of the three *A. baumanii* strains were XDR, and the rest were MDR. All of the *K. pneumoniae, S. aureus*, *Proteus* spp. were MDR. Only the two *E. coli* strains had a better antibiotic susceptibility. The positive samples were collected between 2 and 9 days (median 3 days) after ICU admission.

More than half (51.1%) of the patients developed oral candidiasis during their stay in the ICU, but only 5.4% had *C. difficile* colitis.

## 4. Discussion

There are concerns that atb overuse in COVID-19 patients will drive antimicrobial resistance. Data from the first two years of the pandemic show that, in Europe, 63.1% of these patients received at least one atb during their illness [7]. Data on antimicrobial stewardship activities on severe/critical COVID-19 patients are limited but needed in order to limit atb overuse in treating a viral disease and/or its complications.

Recent data from a large multicenter study [20] show that the most frequent bacterial infection acquired in the ICU by COVID-19 patients was pneumonia (14%) followed by non-catheter-associated bacteremia, despite the fact that 85% of them received antibiotics (35% azithromycin). The rates of bacterial co-infection among ICU COVID-19 patients are higher than in similar non-SARS-CoV-2-infected patients prior to the pandemic: 44–54% [20,21,22] vs. 8.3% [23]. In our patients, secondary bacterial pneumonia was diagnosed in 32.6% of patients and bacteremia was evidenced in 2.7% of them.

A significant proportion of our patients had one or more risk factor for severe COVID-19 [24,25,26]. Most of them (56%) were more than 65 years of age and male (53.3%) with three or more co-morbidities (61.4%). This frailty of our patients and the frequent use of corticosteroids (97.8% of patients) and immune-modulating drugs such as Tocilizumab (34.2% of patients) or Anakinra (52.7% of patients) could partially explain the widespread use of potent antibiotics in our patients. Tocilizumab or Anakinra use in COVID-19 patients has been linked to an increased risk of bacterial co-infection, but the results of several studies vary greatly (from 43% to none) [27,28,29,30,31,32]. The use of steroids in the ICU has been linked with the risk of developing bacterial co-infections, with MDR microorganisms according to the study of Morris et al. [20], and with bacterial pneumonia [33], but several other studies did not find such associations [34,35,36].

Following the first months of relative confusion, most countries or healthcare facilities locally developed guidelines for the treatment of COVID-19 according to the information and resources available at that moment. In Romania, the first national guide was published in March 2020, as a Health Ministry’s order [37]. It stated that, outside the ICU, the bacterial co-infection in SARS-CoV-2 infected patients is rare and strong clinical, radiological, and laboratory parameters should be used before starting an antibiotic treatment in these patients; for the mechanically-ventilated ICU patients, the frequency of associated pneumonia (VAP) is supposed to be low and the antibiotic therapy should be adapted to the local resistance profile. Several updates of these guidelines have been published in 2020 [38] and 2021 [39], each stating that antibiotics should not be given to COVID-19 patients, except in selected cases, and that the VAP risk is below 20%, even after 3 weeks of assisted ventilation.

As previously reported by other authors [6,7], a significant proportion of our patients had received oral atb before their hospitalization—42.9%—more than half of them without a medical prescription. This self-medication reflects a poor atb sales control in ambulatory settings in our region and a high degree of misinformation and fear in the community.

Due to some early hopes [40] and wide media coverage, azithromycin has been largely used in COVID-19 patients. In our study, it was the most-used antibiotic in ambulatory settings, with 18% of our patients receiving it before the admission to the hospital. Its use did not significantly decrease in 2021 patients (19 vs. 16.5% in 2020, *p* = 0.67), despite the fact that several large, randomized trials discouraged its employment in COVID-19 patients [41,42].

Five of the seven atb used in ambulatory settings were included in the Watch category of the 2021 AWaRe classification (azithromycin, cefixime, ciprofloxacin, levofloxacin and clarithromycin); 30.4% of our patients received this type of atb before hospital admission.

A total of 68% of the patients admitted in the ID ward, before the ICU, received an atb, and Ceftriaxone was the most-used one (54% of patients), despite the national guidelines discouraging the use of atb in this category of patients [38,39]. The overuse of Ceftriaxone in this type of patients was previously reported [43,44,45].

Some initial guidelines regarding COVID-19 patients with critical forms of disease and hypoxic respiratory failure requiring mechanical ventilation suggested empiric antimicrobial treatment that should be evaluated for de-escalation on a daily basis [46,47]. Accumulating data suggest that bacterial infection at hospital admission is infrequent in COVID-19 patients (4.4% of patients (95%CI 3.0–6.4%; n = 125,212) and only 8.2% of the patients (95%CI 6.3–10.7%) developed a secondary infection while in a general hospital ward [48]. On the other hand, at ICU admission, 15.5% (95% CI 10.5–22%) of patients had a bacterial coinfection and 41.9% (95%CI 29.5–55.4) of these patients later developed such an infection [48].

The latest WHO guideline for the management of COVID-19 recommends early and appropriate empiric antimicrobial therapy for patients with severe disease [49]. It should be based on the clinical diagnosis, local epidemiology, and susceptibility data as well as national treatment guidelines [49]. All of our ICU patients received at least one antibiotic, but an association of two was the most common occurrence (35.9%). The most common association was Imipenem (Watch group) and Linezolid (Reserve group)—51% of patients. In 19% of patients, due to de-escalation or escalation, more than three atb (maximum of six) were serially used. As per guidelines, changes in the atb regimens were initiated on clinical grounds (8.8%), worsening of Rx findings (9.8%), or increases in the number of leucocytes and CRP (63%).

We did not find an association between the patients’ comorbidities (assessed by the Charlton score) or the outcome of the patient and the number of the received antibiotics. More than one-third of the evaluated patients had elevated procalcitonin levels at ICU admission, and this could be another argument for the administration of antibiotics in our patients [50], although some studies associate this elevated level with disease severity and not bacterial co-infection [51,52]. Most inflammatory markers were elevated at ICU admission (IL-6, ferritin, CRP, number of leukocytes or neutrophils), but none were associated with the disease outcome. The neutrophil-to-lymphocyte ratio (NLR) at ICU admission was only slightly higher in survivors (13.9 vs. 11.6, *p* = 0.46); this biomarker had a high predictive value at hospital admission or when the leukocytes were at their maximum value, but its value decreased with time [53,54,55] (our median interval between symptoms onset and ICU admission was 8 days).

At the beginning of the pandemic, a large meta-analysis [7] estimated the frequency of bacterial co-infection in COVID-19 patients at 8.6% (95% CI 4.7–15.2%) from 31 studies. Most newer studies deliver similar estimates (between 5 and 8%) [56,57,58]. A higher frequency was reported from ICU patients (56%) [59], with the most common isolates being *Enterobacter* spp., *Staphylococcus aureus* and *Klebsiella* spp. In our study, strong clinical and/or biological and radiological findings suggesting bacterial co-infections were present in 41.8% of patients, but bacteriological confirmation was obtained in only 9.8% of patients; the most frequent isolates were of *Pseudomonas aeruginosa, A. baumanii* and *K. pneumoniae;* they were acquired during the ICU stay and had a poor antibiotic susceptibility (6 XDR and 12 MDR). The involvement of antibiotic-resistant bacteria in ICU patients is not uncommon [60] and has been previously reported also in COVID-19 patients [61], and our study seems to support these findings. By empirically using newer, broad-spectrum atb at ICU admission in critically ill SARS-CoV-2-infected patients, we are trying to avoid or to treat such resistant pathogens, but without clear atb stewardship rules, we might also promote the apparition of resistant strains.

The fatality rate of COVID-19 ICU-treated patients varied extensively in the world, being influenced by the SARS-CoV-2 variant, geographical and socio-economic conditions, type of available therapies, patients’ comorbidities, and other factors. For the two first years of the pandemic, Kloka et al. [62], in a large Germany-based study, reported an overall mortality of 33.4%, Armstrong [63] found a fatality rate between 35 and 41% in an England-based study, and the mortality rate was shown to be as high as 72.5% in Mexico [64]. In our study, the fatality rate was 68.5%, higher than in other European countries. We observed that it was slightly higher in patients who received three or more antibiotics during their illness—74.2% vs. 63.2%.

*C. difficile* infection (CDI) is usually associated with broad-spectrum antibiotic use and was previously reported in COVID-19 patients, its frequency varying from 0.4 to 10% according to several studies [65,66,67]. It worsens the disease course, increasing the need for mechanical ventilation (compared with no diarrhea) in ICU patients [67]. CDI in such patients significantly decreases their survival rates [67,68]. In our study, 5.4% of patients had CDI, similar to Maslennikov’s findings [67], and the survival rate in these patients was only 20% (higher, but not significantly, than in patients without CDI).

Our study has several limitations, starting with its retrospective, observational nature, single center design, the lack of a comparator pre-pandemic group; the follow up of the patients ended with the hospital discharge and some complications related to the atb usage could have occurred later (e.g., CDI).

## 5. Conclusions

All of our COVID-19 patients received at least one (usually more) broad-spectrum antibiotic while in the ICU, motivated in most cases only by indirect clues for a possible bacterial infection, and this trend did not decrease in patients in 2021 (compared to 2020). Although some guidelines [46,47,49] recommend early atb in severe cases of SARS-CoV-2 infection, strict antibiotic stewardship principles, surveillance, and de-escalation of the treatment are needed in ICU patients in order to avoid unjustified Watch and Reserve Group atb consumption, the appearance of bacterial resistance, or other side effects (such as fungal infection or CDI).

## Figures and Tables

**Table 1:** Demographics and patient comorbidities.

COVID 19 ICU Patients	N = 184
Median Age, years, (min, max)	68 (34–93)
Male (%)	53.3
Primary comorbidities	N (%)
HTA	99 (53.8)
Cardio-vascular diseases (other)	63 (34.2)
Diabetes	52 (28.3)
Chronic hepatitis	11 (6)
Cancer	13 (7.1)
Auto-immune diseases	6 (3.3)
Chronic kidney disease	10 (5.4)
Dementia	7 (3.8)
COPD	4 (2.2)
Other causes of immunosuppression	15 (8.2)
Obesity o BMI 30–34.9 kg/m^2^ o BMI 35–39.9 kg/m^2^ o BMI > 40 kg/m^2^	40 (21.7) 33 (17.9) 18 (9.8)
Charlton comorbidity index—median (min, max)	3 (0–8)
No of co-morbidities/patient 0 1 2 3 >3	14 19 38 51 62

**Table 2:** Recorded causes of antibiotic changes in the COVID 19 ICU patients.

Cause	No. Patients	%
Clinical deterioration	8	8.7
Rise in no of leucocytes and CRP	58	63.0
Microbiological results	10	10.9
*Clostridioides difficile* infection (CDI)	4	4.3
Unknown	3	3.3
Worsening of Rx findings	9	9.8

**Table 3:** Number of antibiotics received by the patients during their disease.

Number of Antibiotics	No. Patients	%
1	29	15.8
2	66	35.9
3	54	29.3
>3	35	19.0

**Table 4:** Mean levels of some inflammatory markers according to the patient’s outcome.

Outcome	IL-6 (pg/mL)	Ferritin (microg/L)	Fibrinogen (g/L)	CRP (mg/L)	Leukocytes No.	Neutrophils (%)
death	361.2	1610.5	4.9	119.0	11,046.5	83.5
survivors	494.0	1570.7	4.8	117.8	10,559.3	84.8
*p*	0.68	0.91	0.8	0.9	0.6	0.4

## Data Availability

All data generated or analyzed during this study are included in this article. Further enquiries can be directed to the corresponding authors.
